# Interneuron Development Is Disrupted in Preterm Brains With Diffuse White Matter Injury: Observations in Mouse and Human

**DOI:** 10.3389/fphys.2019.00955

**Published:** 2019-07-30

**Authors:** Helen B. Stolp, Bobbi Fleiss, Yoko Arai, Veena Supramaniam, Regina Vontell, Sebastian Birtles, Abi G. Yates, Ana A. Baburamani, Claire Thornton, Mary Rutherford, A. David Edwards, Pierre Gressens

**Affiliations:** ^1^Department for Comparative Biomedical Sciences, Royal Veterinary College, London, United Kingdom; ^2^Department of Perinatal Imaging & Health, Centre for the Developing Brain, School of Biomedical Engineering and Imaging Science, King’s College London, London, United Kingdom; ^3^Université de Paris, NeuroDiderot, Inserm, Paris, France; ^4^School of Health and Biomedical Sciences, RMIT University, Melbourne, VIC, Australia; ^5^Department of Neurology, University of Miami, Miller School of Medicine, Miami, FL, United States; ^6^Department of Pharmacology, University of Oxford, Oxford, United Kingdom

**Keywords:** parvalbumin, perineuronal nets, neuroinflammation, mouse, human

## Abstract

Preterm brain injury, occurring in approximately 30% of infants born <32 weeks gestational age, is associated with an increased risk of neurodevelopmental disorders, such as autism spectrum disorder (ASD) and attention deficit hyperactivity disorder (ADHD). The mechanism of gray matter injury in preterm born children is unclear and likely to be multifactorial; however, inflammation, a high predictor of poor outcome in preterm infants, has been associated with disrupted interneuron maturation in a number of animal models. Interneurons are important for regulating normal brain development, and disruption in interneuron development, and the downstream effects of this, has been implicated in the etiology of neurodevelopmental disorders. Here, we utilize postmortem tissue from human preterm cases with or without diffuse white matter injury (WMI; PMA range: 23^+2^ to 28^+1^ for non-WMI group, 26^+6^ to 30^+0^ for WMI group, *p* = 0.002) and a model of inflammation-induced preterm diffuse white matter injury (i.p. IL-1β, b.d., 10 μg/kg/injection in male CD1 mice from P1–5). Data from human preterm infants show deficits in interneuron numbers in the cortex and delayed growth of neuronal arbors at this early stage of development. In the mouse, significant reduction in the number of parvalbumin-positive interneurons was observed from postnatal day (P) 10. This decrease in parvalbumin neuron number was largely rectified by P40, though there was a significantly smaller number of parvalbumin positive cells associated with perineuronal nets in the upper cortical layers. Together, these data suggest that inflammation in the preterm brain may be a contributor to injury of specific interneuron in the cortical gray matter. This may represent a potential target for postnatal therapy to reduce the incidence and/or severity of neurodevelopmental disorders in preterm infants.

## Introduction

While preterm birth has a multifactorial etiology, it is widely recognized as being precipitated by pro-inflammatory events (reviewed by Hagberg et al., 2015), and these vulnerable infants are at risk of further exposure to inflammation and infection. As such, in preterm born infants, the severity and duration of inflammation highly correlates with long-term outcome (Kuban et al., 2015). Inflammation is also a risk factor in the development of neurodevelopmental disorders (Hagberg et al., 2012; Jiang et al., 2018), such as autism spectrum disorder (ASD), attention deficit hyperactivity disorder (ADHD), childhood epilepsy and disorders of learning, and cognition and emotional development. Given the associations, it is possibly unsurprising that up to 30% of preterm born infants are diagnosed with a neurodevelopmental disorders in childhood (Marlow et al., 2005; Wood et al., 2005; Johnson et al., 2010; Franz et al., 2018). Neurodevelopmental disorders have a substantial effect on the quality of life of affected individuals and their families, but we have limited options to improve the brain health of these infants. The development of therapies is hampered by the fact that the neuropathology of developmental disorders is as mixed as the diagnosis, varying both between and within specific clusters of disorders.

Specifically in preterm born infants, patterns of white matter injury, and increasingly of a gray matter injury, are recognized and associated with poor outcome. In contemporary cohorts of preterm infants, white matter injury includes periventricular leukomalacia in the most severe cases and more commonly diffuse white matter injury (Counsell et al., 2003; Back et al., 2007; Buser et al., 2010). The scale of white matter injury correlates with the severity of the outcome for preterm born infants (Counsell et al., 2008; Keunen et al., 2017; Tusor et al., 2017). Diffuse white matter injury has been successfully modeled in mice and sheep by recapitulating the exposure to early-life inflammation seen in preterm born infants (Mallard et al., 2003; Stolp et al., 2005; Dean et al., 2009; Favrais et al., 2011), though chronic hypoxia can result in similar pathology (Back et al., 2006), supporting a multifactorial etiology. Improvements in imaging modalities have made it possible to study the ultrastructure of the gray matter. Concomitantly, there has been a reduction in the most severe forms of white matter injury allowing the nature of subtle gray matter deficits to be probed in more detail. The microstructural pathology in the gray matter of preterm infants is still an on-going study (Ajayi-Obe et al., 2000; Boardman et al., 2006; Ball et al., 2013; Batalle et al., 2019), but it is clearly linked with the later development of cognitive disorders (Kersbergen et al., 2016; Lean et al., 2017). However, inflammation, synaptic dysfunction, and altered gamma-aminobutyric acid (GABA) signaling are frequently identified as underlying causes and mechanisms of injury in neurodevelopmental disorders (Pardo and Eberhart, 2007; Pinto et al., 2010; Deidda et al., 2014; Coelewij and Curtis, 2018; Dark et al., 2018). This suggests that synaptic dysfunction and altered GABA signaling are valid candidates to mediate the neurodevelopmental disorders associated with encephalopathy of prematurity.

Interneurons make up approximately 20–30% of cells within the cortex (slightly higher in primates than mice; Jones, 2009) and provide inhibitory balance for the excitatory pyramidal neurons by connecting cortical layers and cortical regions *via* interactions with glia, the vasculature, and other neurons (Cossart, 2011; Marin, 2012, 2013; Barber et al., 2018). Interneurons are primarily born within the medial and lateral ganglionic eminence during embryonic development and migrate to the cortex during the early postnatal period, where they mature over the first few weeks of life in mice (Cossart, 2011), equivalent to early childhood in humans. This maturation process includes differential expression of subtype markers (see below), maturation of dendritic arbors, and synpases, as well as changes in synaptic activity, and contacts, and transient networks (Butt et al., 2005, 2017; Cossart, 2011; Marques-Smith et al., 2016). There is a complex array of interneuron subtypes based on their morphological, electrophysiological, and molecular signatures (Kelsom and Lu, 2013). Major populations of interneurons in the cortex include those immunopositive for somatostatin (SST), calbindin (CalB), calretinin (CalR), and parvalbumin (PV). Changes in interneurons are termed an interneuronopathy, and deficits in each of these cellular subtypes have been associated with neurodevelopmental disorders (Marin, 2012), including those prevalent in preterm born children. There is currently little known about the effect of prematurity on cortical interneurons, although a recent study by Panda et al. (2018) has shown a general decrease in GABAergic (glutamate decarboxylase, GAD, positive) interneurons in the cortex, primarily driven by a decrease in parvalbumin-positive interneurons in the upper (layers II–IV), and to a lesser degree, lower (layers V and VI) cortex (Panda et al., 2018). However, this study focused on the effect of early or later prematurity and did not specifically study cases of encephalopathy of prematurity compared with controls. Changes in parvalbumin-positive interneurons in particular are commonly reported in studies of patients with neurodevelopmental disorders or in associated animal models (Kataoka et al., 2010; Gandal et al., 2012; Bitanihirwe and Woo, 2014; Barnes et al., 2015; Filice et al., 2016; Hashemi et al., 2018; Vogt et al., 2018). Parvalbumin knockout mice have ASD-like behavioral symptoms (Wohr et al., 2015). Additionally, knockout of metabotropic glutamate receptors (mGluR5) in parvalbumin interneurons results in specific memory deficits, altered sensory motor gating, and increased compulsive-like behaviors (Barnes et al., 2015). Similarly, maternal immune activation, a common experimental model for ASD, reduces parvalbumin inhibitory activity on pyramidal neurons, resulting in defects in attentional shifting (Canetta et al., 2016). Interneurons, including parvalbumin-positive interneurons, have a specialized area of extracellular matrix (chondroitin sulfate proteoglycans) surrounding them, called a perineuronal net. It has been shown in the past years that this net forms and enlarges during development, representing a marker of a functionally mature neuron. The perineuronal net plays a role in regulating plasticity, and deficits are associated with epilepsy induced plasticity (Galtrey and Fawcett, 2007).

Here, we aim to assess whether there is a subtype specific disruption in interneuron maturation in our population of preterm infants with diffuse white matter injury compared with age-matched controls. Further to this, in mice exposed to IL-1β-induced inflammation, which produces diffuse white matter injury (Favrais et al., 2011; Krishnan et al., 2017), we will assess the long-term trajectory of interneuron development and the consequences for wider cortical maturation.

## Materials and Methods

### Human Postmortem Tissue

Written informed parental consent was acquired according to the National Health Services (NHS) UK guidelines and study ethics were obtained from the National Research Ethics Services (West London), UK (ethics number: 07/H0707/139; Postmortem Magnetic Imaging Study of the Developing Brain). Thirteen extremely preterm postmortem brains (<30 weeks gestational age, 1 female/12 male) of vaginally delivered neonates were used in this study and obtained from the Perinatal Pathology Department, Imperial Health Care Trust, London, UK. The primary cause of death for each case was assessed by a pathologist. Brain tissue blocks from these cases had a postmenstrual age (PMA) range from 23^+2^ to 30^+0^ weeks, calculated by GA (at birth), plus age at death (PMA range for each cohort: 23^+2^ to 28^+1^ for non-WMI cases, 26^+6^ to 30^+0^ for WMI cases, *p* = 0.002). The details of each case are summarized in Table 1. Amniotic fluid infections were identified in most cases; however, no cases had identifiable vascular thrombosis or leptomeningitis. From postmortem examination, brains were assessed macroscopically and microscopically. Seven cases showed no significant brain pathology, these were used as non-neuropathologic controls (no WMI cases). Six brains had evidence of diffuse (non-cystic) white matter injury (WMI cases) including white matter gliosis and focal lesions.

**Table 1:** Summary of clinical information of human postmortem cases.

Group	Sex	GA at birth (weeks)	Postnatal age	PMA at death (weeks)	Clinical context	Neuropathology
**No white matter injury**						
1	M	23 + 2	5 min	23 + 2	Ass IUGR, oligohydramnios, congestive heart failure	
2	M	23 + 6	9 h 51 min	23 + 6	Ass IUGR (twin)	Leptomeninges congested with focal hemorrhages
3	F	24 + 1	4 h 40 min	24 + 1	Extreme prematurity, congestive heart failure	
4	M	24 + 2	IUD/stillbirth	24 + 2	Constricted umbilical cord, congestive heart failure	
5	M	25 + 3	21 h 7 min	25 + 3	Ass IUGR (twin)	Odematous brain with transtentorial herniation of the unci
7	M	26 + 2	43 h	26 + 3	TTT, Ass IUGR, pulmonary hemorrhage	
8	M	28 + 1	<1 h	28 + 1	Oligohydramnios, lung hypoplasia, congestive heart failure	
**Non-cystic white matter injury**						
1	M	26 + 5	1 days 7 h 52 min	26 + 6	Ass IUGR, oligohydramnios, congestive heart failure	PVWM injury/pathology in PVWM at angles of the lateral ventricle
2	M	24 + 6	16 days 19 h 10 min	27 + 0	AAAFI, PPROM, acute necrotizing pneumonia	PVWM injury/pathology in PVWM at angles of the lateral ventricle
3	M	24 + 0	5 weeks 1 day 8 h 25 min	29 + 1	AAAFI, retroplacental hemorrhage	PVWM injury/pathology in PVWM at angles of the lateral ventricle
4	M	29 + 3	11 h 17 min	29 + 3	TTT, AAAFI, hydrops fetalis	GM/IVH
5	M	27 + 5	12 days	29 + 4	Ass IUGR, necrotizing entercolitis, uterine constraint/oligohydramnios	PVWM injury/pathology in PVWM at angles of the lateral ventricle
6	M	26 + 0	1 min	30 + 0	Necrotizing enterocilitis	Patchy WM gliosis

*Ass IUGR, asymmetric interauterine growth restriction; AAAFI, acute ascending amniotic fluid infectin; GM, germinal matrix; IVH, intraventricular hemorrhage; PPROM, preterm premature rupture of the membranes; PVMW, periventricular white matter; TTT, twin-twin transfusion; WM, white matter*.

As previously reported (Supramaniam et al., 2013; Vontell et al., 2013) after postmortem, whole brains were fixed with 4% formalin for 5–7 weeks, depending on size. The whole brains were sliced by a pathologist, and tissue blocks were processed on a Bright Tissue Processor (Bright Instrument Co. Ltd.). Paraffin-embedded tissue blocks of the frontal lobe at the level of the caudate (i.e., anterior to Ammon’s Horn) were sectioned at 6 μm using a Leica RM2245 microtome (Leica Microsystems Ltd.).

### Animal Model

All animal procedures were approved by the UK Home Office according to the regulations in the Animal (Scientific Procedures) Act (2012), and the King’s College London (KCL) Animal Welfare and Ethical Review Board (AWERB; PPL 70/8376). Inflammation-associated brain injury of the preterm born infant was modeled in mice by exposing them to systemic inflammation from postnatal day 1 (P1) through to P5; P0 is the day of birth, as previously reported (Favrais et al., 2011). P1–P5 is approximately equivalent to the period of 23–32 weeks gestation for brain development in the human pregnancy, based on a mixture of myelination and cortical development processes (Clancy et al., 2001, 2007; Semple et al., 2013). Pregnant CD-1 mice were purchased from Charles Rivers and transferred to the KCL Biological Services Unit (BSU) at embryonic day (E) 16 of pregnancy. Animals were housed separately in individually ventilated cages with food and water available *ad libitum*, in a temperature controlled environment with a 12 h light-dark cycle. This injection paradigm only produces consistent and reproducible diffuse white matter injury in male mice (Favrais et al., 2011), compared with female mice. Therefore, following birth, female pups were culled by cervical dislocation, and male pups were randomly divided into litters for saline or IL-1β treatment (typically numbering four to seven pups per “new” litter). Each pup received a 5 μl intraperitoneal (i.p.) injection twice daily from P1 to P4 and a final injection in the morning at P5 (Favrais et al., 2011). IL-1β (R&D Systems) was diluted in saline to a working concentration of 8 ng/μl (for a final dose of 10 μg/kg/injection). Animals remained housed in litter groups until weaning (P21), when they were group housed (3–4/cage). At P5 (6 h post final injection), P10 (approximate time of six layered cortex formation), P40 (when interneuron markers are mature), and P60 (early adulthood) randomly selected pups from each litter were killed by terminal anesthesia (i.p. pentobarbitone overdose) and perfused with saline followed by cold 4% paraformaldehyde. The brain was dissected out of the skull and immersion fixed in Bouin’s solution (Sigma). Fixed tissue was washed and dehydrated through graded alcohol (progressing from 50 to 100%) and embedded in paraffin wax (Sigma, UK). Coronal 6-μm thick consecutive sections were cut and placed on microscope slides, with three sections at an interval of approximately 250 μm per slide.

### Immunohistochemistry and Microscopy

As previously reported (Vontell et al., 2013), postmortem human sections underwent routine paraffin removal and rehydration, then were placed in 3% hydrogen peroxide to quench endogenous peroxidase activity, and immersed in preheated 10 mM citric acid with 0.1% Tween-20 (VWR International Ltd.) for 30 min and cooled at room temperature for 20 min. Sections were blocked with 5% normal goat or horse serum (as appropriate, based on the secondary antibody host species) and primary antibodies were incubated overnight at 4°C; concentrations below. The next day, biotinylated secondary antibodies (1:200, Vector Laboratories) goat anti-rabbit, goat anti-rat, or horse anti-mouse were incubated for 1 h at room temperature, then with avidin-biotin complex (ABC, 1:200, Vector Laboratories, UK) for 1 h. The reactions were visualized with 3,3′-diamino-benzidine (DAB; Sigma-Aldrich Company) for 10 min. Sections were then dehydrated, cleared in xylene, and cover-slipped. Primary antibodies used to identify all neurons in the developing human cortex were mouse anti-HuC/HuD (1:500, Life Technologies) and interneuron markers; mouse anti-calretinin (CalR; 1:100, Millipore), mouse anti-calbindin D-28 (CalB; 1:100, Sigma), rabbit anti-parvalbumin (PV; 1:500, Abcam), rat anti-somatostatin (SST; 1:50, Abcam), rabbit polyclonal neuropeptide Y (NPY; 1:5,000, Abcam). Routine H&E staining was also performed on this cohort of brain samples to assess for gross neuropathologies.

Mouse tissue was processed as described above and stained as previously reported (Stolp et al., 2011). Mouse anti-CTIP2 (1:400; Abcam) was used to identify neurons in the developing cortical plate in the mouse (Stolp et al., 2011; Garcez et al., 2018). Interneuron populations were stained with one of rabbit anti-Parvalbumin (PV, 1:200, Abcam), rabbit anti-Calretinin (CalR, 1:200, Swant), rat anti-Somatostatin (SST, 1:100, Abcam), rabbit anti-Neuropeptide Y (NPY, 1:100, Abcam), mouse anti-Calbindin (CalB, 1:100, Sigma), mouse anti-Reelin (1:500, Calbiochem), rabbit anti-Vasoactive Intestinal Peptide (VIP, 1:100, Abcam). Perineuronal nets were identified using biotinylated *Wisteria floribunda* Lectin (WFL, 1:200, Vector). Sections were incubated in the appropriate biotinylated or fluorescently-tagged secondary antibody: biotinylated goat anti-rabbit or mouse (1:200, Vector); donkey anti-mouse-488; donkey anti-rabbit-488/546 (1:400, Invitrogen) or streptavidin-488 (1:400, Invitrogen). All antibodies and lectins were diluted in 1% donkey serum in phosphate-buffered saline with 1% Tween-20. Sections with fluorescent secondaries were incubated with DAPI for 5 min (4′,6-diamidino-2-phenylindole, 1:1,000, Sigma-Aldrich) and mounted with ProlongGold (Thermo Fisher Scientific).

A subset of brains were processed for Golgi staining using the FD Rapid GolgiStain Kit (FD Neurotechnologies Inc.), following the manufacturer’s instructions. Briefly, brain tissue was washed in dH_2_O immediately following collection and then incubated in impregnation solution for 2 weeks. After this period, the samples were incubated in Solution C for 3 days before tissue sections were cut with a Vibratome (Leica) at 100 μm thickness, mounted in slides, and dehydrated through ethanol and xylene before mounting with DPX (Sigma).

### Image Analysis and Statistics

Human tissue sections were visualized with bright-field microscopy, using a light microscope (Leica DM6000B, Leica Microsystems Ltd.), CCD color video camera (Leica CTR6000, Leica, UK), equipped with a motorized stage for automated sampling (MicroBrightfield Inc.). Cell number was assessed using optical fractionator stereological software (Stereo Investigator v8.27, MicroBrightfield Inc.). Contours were drawn around the frontal cortex magnified by a 5× objective (0.0426 mm^2^), with an average area for each contour of 1 mm^2^. Counting was performed at the magnification provided by the 40× objective, from multiple counting sites throughout the contour, to allow unbiased sampling of the frontal cortex and an estimate of cell density. The number of counting sites was varied for each cell type, due to differences in density (determined from preliminary assessments of cell number); overall, an average of 35–45 counting sites were used for each contour (Vontell et al., 2013, 2015). Three contours were assessed for each brain region of interest (cortex and subcortical white matter, therefore, approx. 3 mm^2^ assessed in total for each) and averaged to produce the estimated cell density for each region. Neurite lengths were assessed in positively stained somatostatin and neuropeptide Y neurons from images obtained with a ×40 objective. An average of seven cells were assessed per case, using ImageJ software (Schneider et al., 2012).

Mouse tissue sections were imaged with a Leica SP5 confocal microscope (P5, P10, and P40 fluorescently stained mouse tissue) or Leica DM4000 upright microscope (P40 mouse tissue). For cell counts, upper (II–IV) and lower (V and VI) layers were delineated as a region of interest for each image, and the immunoreactive cells counted manually, by a blinded observer, using the ImageJ cell counter tool. For the parvalbumin/perineuronal net counts, multiple markers were used so that single- and double-labeled neurons could be counted together in a single region of interest. Images were taken from the area of the somatosensory cortex identified by the presence of the barrel cortex, and measurements were averaged from both the medial (M1) and lateral (S1BF) cortex (Paxinos and Franklin, 2012), from three sections per brain. Therefore, data were averaged from three to six images per brain (tissue was excluded if it was damaged or where the staining had excessively high background or was non-specific), and from three to six brains per treatment and age (P5: *n* = 3–6 both groups; P10: *n* = 5 saline, *n* = 6 IL-1β; P40: *n* = 3 saline, *n* = 5 IL-1β).

Cell counts are presented as mean ± standard deviation (SD). Statistical analysis was performed using Prism 7 (GraphPad). Postmortem human samples were analyzed with an unpaired *t*-test. Grouped data from P5 tissue were analyzed using a two-way ANOVA for treatment and cell population, with *post hoc* analysis performed with Sidak’s multiple comparison test. For P10 and P40 data, grouped data from each cell population were analyzed using a two-way ANOVA for treatment and layer, with *post hoc* analysis performed with Sidak’s multiple comparison test. A *p* <0.05 was considered statistically significant.

Golgi stained neurons, with consistent stain impregnation and isolation from neighboring stained cells, were imaged with a Leica DM6000B microscope for analysis. Stacked images in the z-axis were obtained at ×40 magnification, with a 1 μm interval. Dendritic branching and intersections were quantified and analyzed by Sholl analysis (Gutierrez and Davies, 2007), using ImageJ image processing program. Image stacks were compressed in one 2D image, and the center of the soma was marked for reference. Concentric circles, with radii increasing by 9.06 μm (50 pixels) per circle, were set from the soma center. Multiple neurons (10–15 from multiple slices across the brain) were analyzed from three brains per treatment, and results are presented as the average number (± SD) of dendritic intersections per concentric circle, for each treatment group. Sholl profiles were statistically compared using a two-way ANOVA for treatment and distance from soma.

For spine analysis, dendrites were isolated in images of neuronal cells collected for Sholl analysis. Dendrites chosen were of different lengths, with a substantial portion in the plane of focus, and were representative of spine density and morphology of unselected dendrites. Spine density of selected dendrites was analyzed using Image-Pro Premier image analysis software (MediaCybernectics). Data were blinded, and images were categorized as having a high or low synaptic frequency (41 from saline treated brains, 57 from IL-1β treated) based on the number of synaptic protrusions along the length of the dendrite, and the spaces, if any, between the protrusions (Smrt and Zhao, 2010; Phillips and Pozzo-Miller, 2015). This categorical data were statistically compared using the Fisher exact test, with a *p* <0.05 considered statistically significant.

## Results

### Preterm Born Infants With Diffuse White Matter Injury Had Reduced Number of Calretinin Interneurons in the Cortex and Altered Arborization of Other Interneuron Populations

**Figure 1:**
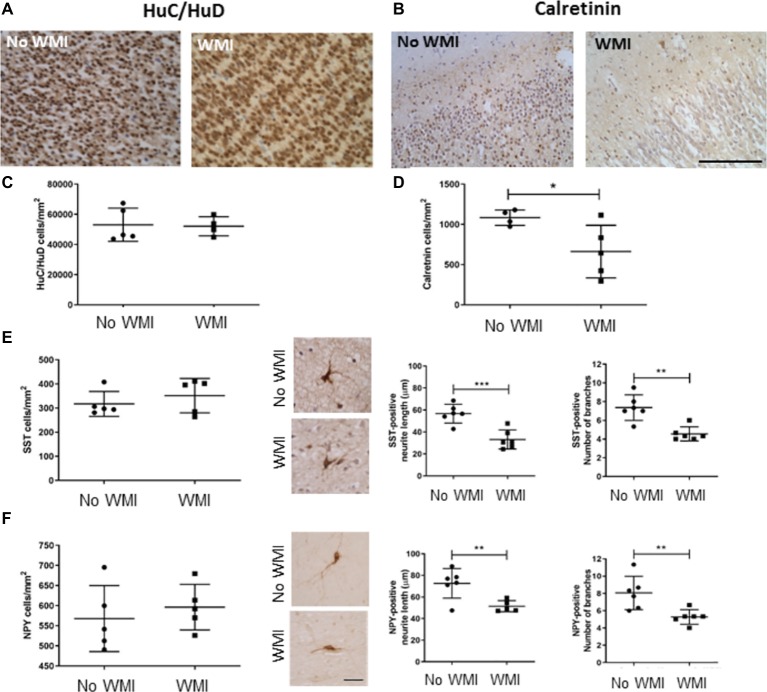
Interneuron development in the cortex and subcortical white matter of the damaged preterm brain is altered compared with age-matched cases without white matter injury. Cell counts were performed in cortical and subcortical white matter in the frontal lobe, anterior to Ammon’s Horn. Total cortical neuronal number was assessed counting HuC/HuD positive neurons **(A,C)**. A significant decrease in Calretinin (CalR)-positive interneurons was observed in the cortex of preterm brains with non-cystic white matter injury (WMI) compared with no WMI controls **(B,D)**. No significant difference in Somatostatin (SST) or Neuropeptide Y (NPY)-positive interneurons was observed in the subcortical white matter. However, SST and NPY labeled interneurons from non-cystic white matter injury showed shorter neurites, with fewer branches **(E,F)**. Data presented as mean ± SD; scale bar = 100 μm **(A–D)**, 25 μm **(E,F)**; ^*^p < 0.05, ^**^p < 0.01, ^***^p < 0.001; SST, somatostatin; NPY, neuropeptide Y; WMI, white matter injury.

Stereological assessment of cell number in the frontal cortex of the developing human brain showed no change in the total number of neurons, identified by HuC/HuD immunoreactivity, with 53,104 ± 11,009 immunopositive cells/mm^2^ found in the control brains (*n* = 5), and 52,120 ± 6,327 cells/mm^2^ in the cortex of the white matter injury cases (*n* = 4). In contrast, there was a significant decrease in the cortical calretinin-immunopositive cells in the white matter injury cases, compared with preterm infants without white matter injury (from 1,084 ± 96 to 663 ± 327 cells/mm^2^, *p* = 0.043, Figures 1A–D). Calbindin- and parvalbumin-positive cells were observed in low numbers in both cases, insufficient for determining statistical significant changes. Somatostatin and neuropeptide Y immunopositive interneurons also occurred much less frequently than calretinin positive cells and were not found in the cortex at this stage of development. However, the cells immunopositive for these markers were found in the subcortical white matter, and these were analyzed for neurite length and branching. There were no statistical differences in the number of cells in either interneuron (SST or NPY) subpopulation between preterm infants with or without white matter injury (*n* = 5 for both). The branching of immunopositive cells was assessed with a modified Sholl Analysis, and there was a significant decrease in the arborization of neurons in both of these interneuron classes. Somatostatin cells in white matter injury cases had shorter leading neurite length (33.2 ± 8.7 μm, *n* = 6, compared with 56.7 ± 8.5 μm in the no WMI cases, *n* = 6; *p* < 0.001) and fewer branches (7.4 ± 1.4 compared with 4.6 ± 0.7, *p* = 0.001). Neuropeptide Y immunopositive neurons also had shorter neurites (51.5 ± 5.2 μm compared with 72.6 ± 13.7 μm in no-WMI group, *n* = 5–6; *p* = 0.01) and fewer branches (5.3 ± 0.85 WMI, compared with 8.1 ± 1.9, *p* = 0.009) in white matter injury cases (Figures 1E,F).

### The Developmental Trajectory of a Number of Interneuron Populations Is Disturbed in a Mouse Model of Preterm Birth

**Figure 2:**
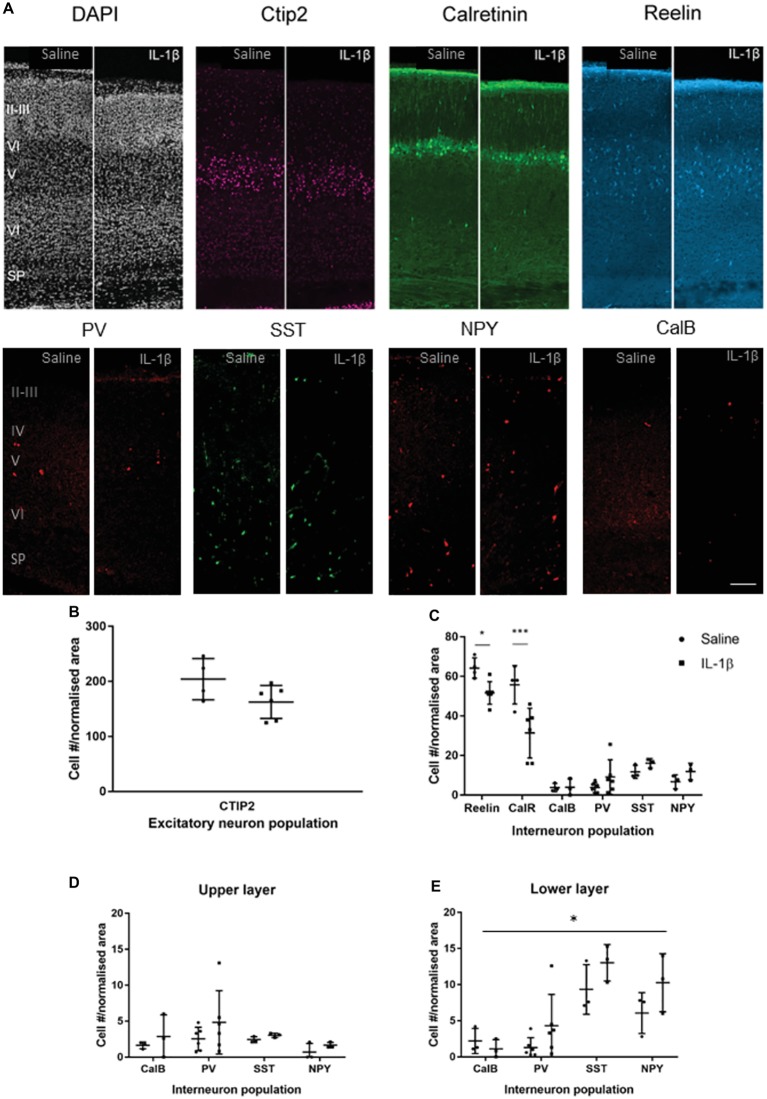
P5 mice with inflammation-induced brain damage also show altered interneuron development across the cortex. Cortical development was assessed based on immunohistochemistry for general microstructure (DAPI), developing cortical neurons (CTIP2) and interneuron subpopulations at P5 **(A,B)**. A significant decrease in Reelin and Calretinin (CalR)-positive neurons was observed in the whole cortex **(C)**, but no change in CTIP2 positive pyramidal neurons **(B)**. Assessment of the less prevalent interneuron populations according to upper (II–IV) or lower (V, VI) cortical layer distribution showed a small but significant increase the majority of interneuron populations in the lower layers (treatment effect, *p* = 0.04, two-way ANOVA) and no significant change in the upper layers **(D,E)**. Data presented as mean ± SD; scale bar = 100 μm. ^*^p < 0.05, ^***^p < 0.001. CalR, calretinin; PV, parvalbumin; SST, somatostatin; NPY, Neuropeptide Y; CalB, calbindin.

In mice with IL-1β-induced inflammation, at the end of the inflammatory exposure (P5), there was no gross alteration in the cortical layering (Figure 2A, DAPI) or cell density. In addition, no statistically significant change in CTIP2 immunoreactive neuron number per cortical area was observed (saline: 204.3 ± 37.4 cells, IL-1β: 162.7 ± 29.8 cells, *p* < 0.086, Figure 2B), suggesting no overall change in the development of the early cortical neurons with injury. There was a significant decrease in the number of reelin-positive (saline: 64.2 ± 5.1 cells, IL-1β: 51.7 ± 5.7 cells, *p* = 0.047) and calretinin-positive (saline: 55.7 ± 9.7 cells, IL-1β: 31.3 ± 12.6 cells, p < 0.001) neurons in the IL-1β treated animals compared with the saline-treated control (Figure 2C). In comparison to these reelin- and calretinin-positive cells, other interneuron populations were present in a much lower number (Figures 2A,C), as would be expected at this early stage of brain development, as the majority of interneuron markers do not fully develop until the second or third postnatal week in the mouse (Cossart, 2011). In general, at this stage of development, the interneuron populations were present in higher numbers in the lower cortical layers (layer V and VI) than in the upper layers (II, III, and IV). Specific analysis of interneuron populations (PV, CalB, SST, and NPY) in the upper layers showed no difference between treatment groups (Figure 2D). In the lower layers, two-way ANOVA analysis showed a significant treatment effect (*p* < 0.043), reflecting a general increase in the same interneuron populations in the IL-1β group (Figure 2E). However, no significant difference was found for individual populations following correction for multiple comparisons.

**Figure 3:**
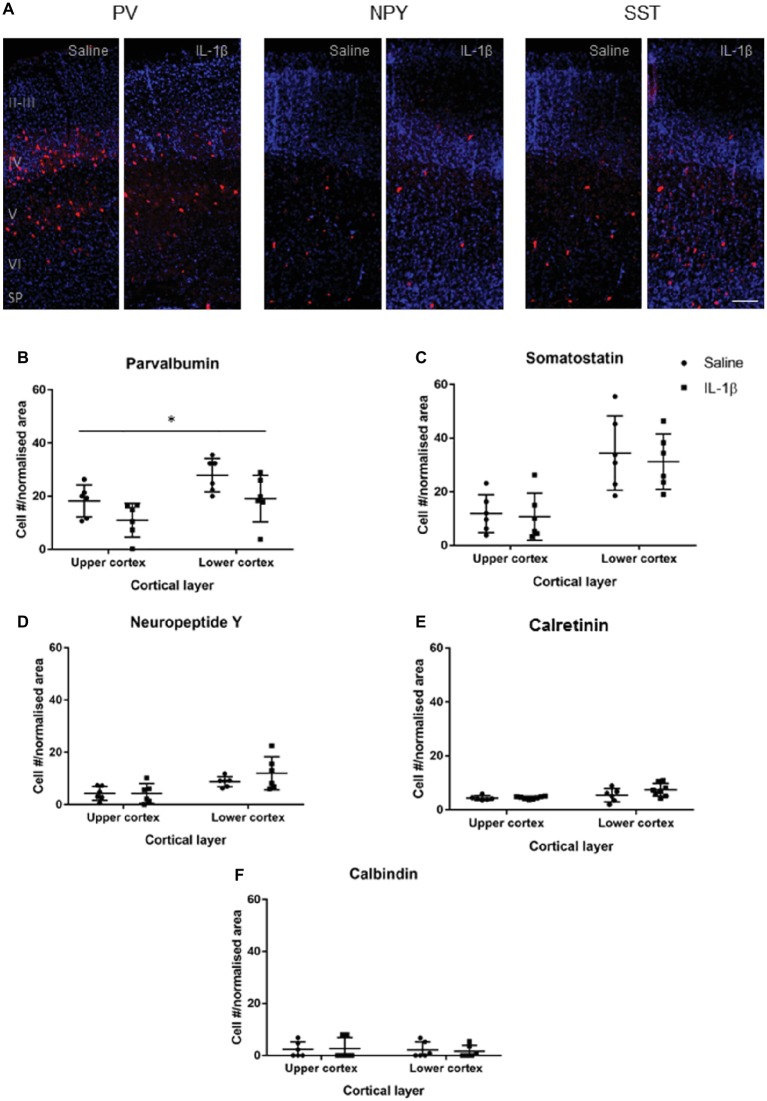
Developmental pattern of interneuron injury changes by P10 in mice with inflammation-induced brain damage. Interneuron populations were assessed again at P10 by immunohistochemistry **(A)** and quantified **(B–E)** for number and distribution through cortical layers. There was a significant decrease in the number of parvalbumin (PV)-positive interneurons across the cortex (**B**; treatment effect, *p* = 0.01, two-way ANOVA), but no change for somatostatin (SST), neuropeptide Y (NPY), calretinin (CalR), or calbindin (CalB, **C–F**). Data presented as mean ± SD; scale bar = 100 μm. ^*^p < 0.05.

At P10 (Figure 3), the somatostatin, neuropeptide Y, calretinin, and calbindin populations were present in greater, though still relatively low, numbers, and there was no significant difference in their presence, or distribution through the cortex between the saline and IL-1β treated groups. Parvalbumin-positive cells were found in significantly greater numbers in layer IV, V, and VI (*p* = 0.005). There was an overall decrease in the number of parvalbumin-positive neurons (*p* < 0.014), with no significant layer effect (Figure 3B).

### Parvalbumin Interneurons Show Long-Term Disruption in Neuronal Number, Arborization, and Association With Perineuronal Nets

**Figure 4:**
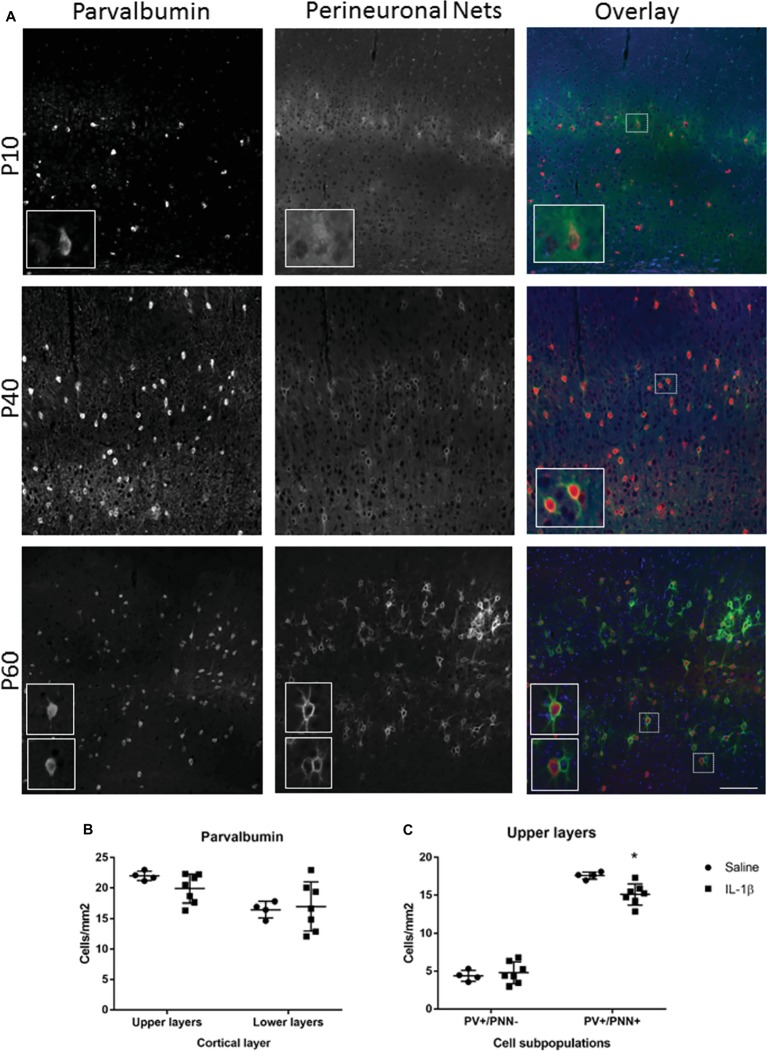
Long-term changes in parvalbumin-positive interneurons and their perineuronal nets extend to P40 in mice with inflammation-induced brain damage. The beginning of perineuronal net formation could be seen in the cortex, primarily in layer IV, from as early as P10 (**A**, top row). The aggregation of the perineuronal nets and their association with the parvalbumin-positive interneurons became more pronounced through development (**A**, lower rows). In the IL-1β challenged mice, there was no gross alteration in perineuronal net formation or in the number of cortical parvalbumin neurons **(B)**. However, there was a significant decrease in the number of PV^+^/PNN^+^ neurons in the upper layers of the cortex **(C)**. Data presented as mean ± SD; scale bar = 100 μm. ^*^p < 0.05. PV, parvalbumin); PNN, perineuronal nets.

**Figure 5:**
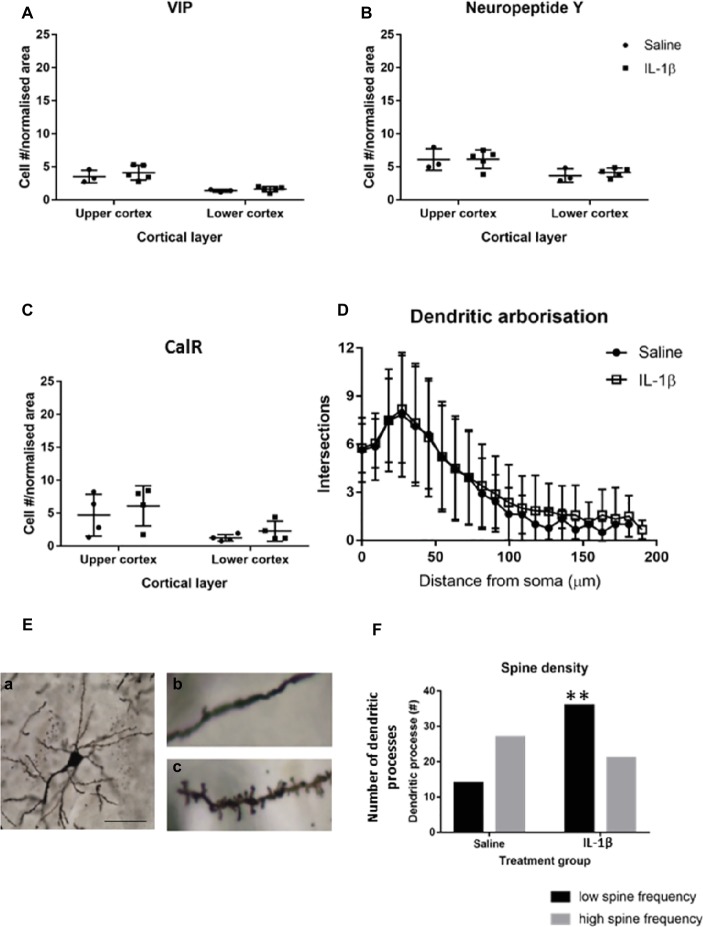
By P40 the majority of interneuron pathology has recovered, but there are long-term changes in dendritic spines associated with the early inflammation-induced brain damage. No difference was seen in the number of neuropeptide Y (NPY), calretinin (CalR), or VIP-positive neurons **(A–C)** at P40. Golgi staining was performed to assess the general morphological development of cortical neurons at this stage. **(E)** Image (**a**) is an example of a well stained cortical neuron used for spine analysis and images (**b,c**) are representative images of spine density on dendrites in the brains of IL-1β and saline treated mice, respectively. There was no significant difference in the dendritic arborisation **(D)** but there was a significant reduction in spine frequency on the dendritic processes in the IL-1β challenged mice **(E,F)**. Data presented as mean ± SD; scale bar = 60 μm for **Ea**, 10 μm for **Eb,c**. ^**^p < 0.01.

To determine whether perturbations in parvalbumin number present at P10 in mice following IL-1β-induced inflammation persisted beyond this early developing period, the number of these neurons was assessed at a later stage of development. At P40, there was a trend toward a decrease in parvalbumin cells in the upper layers of the cortex but not the lower layers (Figures 4A,B). The maturation state of parvalbumin interneurons was assessed by the presence of perineuronal nets, which start to form in a loose association with the parvalbumin neurons in the barrel cortex from P10 in the mouse (Figure 4A). Clear association of the nets with parvalbumin-positive interneurons is visible by P40, which mature further as development continues (P60; Figure 4A; Ueno et al., 2017). When explored in more detail, a statistically significant decrease in parvalbumin-positive neurons that had fully formed perineuronal nets (17.6 ± 0.2 cells/mm^2^ in saline, 15.1 ± 0.5 cells/mm^2^ in IL-1β, *p* = 0.008) was seen specifically in the upper layer of the IL-1β treated animals (Figure 4C). At this age, there was a relatively high proportion of parvalbumin neurons without perineuronal nets (25% in saline), but there was no change in this subpopulation of neurons with treatment (32% in IL-1β group, *p* = 0.8, Figure 4C). Likewise, in brains from both saline and IL-1β treated animals, a small number of perineuronal nets were observed without PV staining (1.5 ± 0.3 cells/mm^2^ in saline and 1.2 ± 0.2 cells/mm^2^ in IL-1β), but the proportions were very low and there was no significant difference between groups. In comparison with these small but significant changes in parvalbumin interneurons, there remained no significant difference in the number of either neuropeptide Y, calretinin, or VIP-positive neurons in the cortex between saline-and IL-1β-treated animals (Figures 5A–C).

### Long-Term Changes in Arborization and Spine Density Are Broadly Observed in Areas of the Cortex With Disrupted Parvalbumin Interneuron Development

Golgi staining was performed to visualize the individual neurons within the cortex, to allow Sholl and spine analysis to be performed. No significant effect of IL-1β treatment was observed on the overall dendritic morphology of neurons at P40 (Figure 5D), but there was a statistically significant shift in the number of dendrites showing a low frequency of spines, with 35% of the spines from Golgi stained cells from IL-1β treated animals showing a low spine frequency compared with 15% in saline-treated tissue (*p* = 0.007, Figures 5E,F).

## Discussion

We have revealed a general decrease in the number of GABAergic interneurons in the cortex of preterm born human infants with diffuse white matter injury, compared with age-matched controls, and similarly in mice with diffuse white matter injury following IL-1β-induced inflammation. This interneuron deficit (reduced numbers and reduced morphological complexity) occurred in the absence of severe injury, as there were no changes in the numbers of neurons in the cortex in the clinical (human) or preclinical (mouse) study. Also, subtypes of interneurons were differentially affected over development indicating population specific vulnerabilities. The early disruption of interneuron populations in our clinical sample is supported by our preclinical evidence that not only did these changes persist but were found together with changes in the excitatory-inhibitory cell balance, spine density, and the density of perineuronal nets. Altogether, these data support the hypothesis that inflammation associated with preterm birth may alter the excitatory-inhibitory balance in the brain. This effect would partly explain the link between exposure to inflammation during development and poor neurodevelopmental outcomes in these infants that lead to life-long functional impairment. However, Canetta et al. (2016) have shown that maternal immune activation at the very beginning of neurogenesis (E9 in the mouse) can also result in parvalbumin-specific pathology, possibly suggesting a particular susceptibility of this cell population to disturbances in normal development. More work will be required to explore this concept.

Interneurons are produced within the medial ganglionic eminence throughout development and migrate to all cortical layers (Butt et al., 2005, 2017). Interneuron fate is genetically coded and follows a clear pattern of migration and maturation, related to the regulatory transcription factors imposed in the ganglionic eminence at the time of cell production (reviewed by Marin, 2012; Kelsom and Lu, 2013). It is postulated that the developmental period during which an injury or perturbation occurs will influence the cell population affected. Injury would also interact with genetic risks, which may affect when phenotypes caused by altered genetic code manifest, explaining the variety of neurodevelopmental disorders associated with similar phenotypes. Animal models allow the progression of injury to be assessed, in comparison with the snapshot of information typically obtained from clinical studies. The utility of the mice with IL-1β-induced inflammation and subsequent diffusion white matter injury used here is that the injury is broadly comparable in nature to that seen in our patient samples. Specifically, this model displays the hallmarks of injury observed in contemporary preterm born infants: hypomyelination, axonopathy, oligodendrocyte dysmaturation and also, reported here and previously, no gross cortical injury evidenced by no change in cortical neuronal number (Favrais et al., 2011; Schang et al., 2014; Krishnan et al., 2017; Rangon et al., 2018). In this model, females have a less severe and more transient phenotype, which is why they were not used in this study (unpublished data), suggesting a bias seen in the animal model similar to that observed in the human population (Marlow et al., 2005). However, it is of course impossible to recapitulate the complex processes leading to injury in the human completely with any model, and we would acknowledge that the differences in the magnitude of the observed changes probably lie in multifactorial injury to the infants.

In this animal model, at the time when the brain is still being subjected to an inflammatory challenge (Krishnan et al., 2017), interneurons appear slightly increased in the cortex of injured animals compared with controls. It is worth noting that most interneuron populations are not properly established in the cortex at this stage (Cossart, 2011), reflected in lower numbers than were found in the adult analysis. As development progresses, and after the injurious stimuli has resolved (at P10, Krishnan et al., 2017), a mild parvalbumin-specific neuronal reduction becomes apparent. Parvalbumin interneurons make up approximately 40% of GABAergic cortical interneurons, which, compared with the less frequent interneuron subpopulations, may make the detection of statistical changes in the cell population easier. Parvalbumin interneurons are fast-spiking interneurons with basket or chandelier morphology that make synaptic connections on proximal dendrites and the soma of target neurons (reviewed by Marin, 2012; Kelsom and Lu, 2013). The cells produce oscillations in the gamma range (30–80 Hz), which is associated with cognitive processes such as memory and attention (Bartos et al., 2007; Canetta et al., 2016). This function may partly explain why parvalbumin knockout mice have an ASD-like phenotype: repetitive behaviors, impaired social interaction and communication, as well as reduced startle response and increased risk of seizure (Wohr et al., 2015).

The severity of injury will also have an effect on outcome, with more severe injury producing larger, more widespread changes. Previous studies of gray matter injury have been performed in human preterm infants with cystic white matter injury, in which there is extensive cell death and reactive gliosis. However, in our human post-mortem cases of diffuse white matter injury, there was no overall change to neuronal number. Possibly unsurprisingly then, compared with our data of limited and cell type-specific changes, studies of gray matter injury in severe cystic cases show more widespread gray matter injury (Andiman et al., 2010; Kinney et al., 2012). As such, our data are an important contribution to understanding how interneuron deficits may impact on neurodevelopmental disorders for contemporaneous cohorts of preterm born infants. The diffuse white matter injury studied here is the predominant form of injury seen in preterm born infants, and cystic cases account for only approximately 5% of neuropathology, based on MRI and ultrasound imaging studies in preterm cohorts (Hamrick et al., 2004; reviewed by Back, 2017).

It must be noted that preterm birth occurs due to a complex, and often undetermined etiology and can be concomitant with other clinical findings, e.g., intrauterine growth restriction (IUGR), which in themselves are also predictive of reduced brain development (Rees et al., 2008; O’Shea et al., 2009; Korzeniewski et al., 2017). The fact that many infants in our cases (both WMI and non-WMI groups) have IUGR may well be a confounder in the injury observed. While the mouse data clearly links interneuron (and white matter) pathology with inflammation, it is likely that there are many contributing factors in preterm brain injury, of which inflammation is only one. This, and the different developmental timetables of mouse and human brains, may also explain the different interneuronopathies identified in this study. Adding to that complexity is the varying age range between the preterm white matter injury and non-white matter injury cohorts examined in this study. From a developmental point of view, the greater PMA of WMI group compared with control should be associated with more visible interneurons in the cortex (through processes of migration and maturation). As a result, we suggest that the significant decrease in interneuron numbers in this group is, if anything, an underestimate of the interneuron injury in preterms with diffuse WMI. However, the longer survival times of these infants does increase the difficulty of defining the etiology of the interneuron injury, as many features of the *ex utero* environment may contribute to the injury severity. For instance, Panda et al. (2018) suggest that deficits in maternal estrogen is a major contributor to interneuron injury. That being said, in the data from the mouse model, when exposure to maternal estrogen (and other factors of the external environment) is constant, there is still a reduction in interneurons.

The significant early decreases seen for interneurons in general in this study are similar to findings recently reported by Panda et al. (2018), where early prematurity in humans was associated with a reduction in parvalbumin-positive interneurons, primarily in the upper cortical layers, and an increase in somatostatin-positive interneurons also in the upper cortex. There was also an overall decrease in glutamate decarboxylase (GAD)-positive neurons, attributed primarily to the changes in the parvalbumin subpopulation. However, a direct comparison with Panda and colleagues is difficult as their cases were compared based on the degree of prematurity rather than on their diagnosis (Panda et al., 2018). This resulted in a comparison of early born infants that had long survival times (up to 3–4 weeks) with later born preterm infants who had survival times of only 2–3 days. The role of postnatal care in interneuron development has been shown in a model of preterm delivery in the baboon, where 14 days of positive pressure ventilation lead a reduction in the numbers of calretinin-positive cells in the visual cortex (Verney et al., 2010). Although this and other confounding factors are almost impossible to remove from a human postmortem study, we are confident that our cases provide evidence more specifically related to the effects of prematurity on interneuron development without the specific effect of postnatal care.

To further assess interneuron number, we investigated their migration through the subcortical white matter in our clinical cases. Reduced morphological complexity was observed for somatostatin and neuropeptide Y immunopositive neurons within the subcortical white matter in this study. As these neurons are still migrating, changes in their morphology may reflect a disruption in migration that will contribute to later reduced interneurons in the cortex, another process associated with neurodevelopmental disorders (Marin, 2013). It is possible that a delay in migration could explain initial reductions in interneuron number in the cortex that lessen or normalize as development continues. Mild models of perinatal injury have recently shown altered neuronal arborization in the sheep subplate (McClendon et al., 2017) and cortex (Dean et al., 2013), though these were not specific to interneurons. These changes in arborization are generally interpreted as a delayed maturation, consistent with diffusion tensor imaging data showing altered diffusion characteristics in the cortex of preterm infants (Ball et al., 2013). These most likely reflect reduced cortical complexity and contribute to a delay in local connection formation (Batalle et al., 2019), which will have downstream effects on subsequent brain development. The mechanism of delayed maturation is, as yet, unclear but could be due to delayed interneuron migration to the correct position within the cortex (as discussed above). Alternatively, this may be due to intrinsic changes that have occurred within the cells as a result of the injury or due to a wider disruption of the network and integration of neurons within it. More work is required to elucidate this process.

Perineuronal nets are a dynamic structure of extracellular matrix proteins that develop around a subpopulation of neurons in the brain. Parvalbumin interneurons are one of the cell types most commonly associated with perineuronal nets, and it has been suggested that these nets modulate the connections between cells and the plasticity to form new connections (Shen, 2018). The presence of perineuronal nets around parvalbumin interneurons regulates Otx2 levels and critical periods of synaptic plasticity between these interneurons and their local networks (Beurdeley et al., 2012). It is unclear in many reports of parvalbumin neuronal loss in clinical cases or animal models of injury/neurodevelopmental disorders whether the deficit is actually in the number of parvalbumin interneurons or in the expression of the parvalbumin protein within the neurons. In a SHANK3 knockout model of ASD, a significant loss of parvalbumin staining was observed in the cortex, but no disruption in the perineuronal nets, implying the presence of “parvalbumin-neurons” but not of the parvalbumin protein (Filice et al., 2016). This phenotype in both SHANK3 and parvalbumin knockout mice suggests that loss of parvalbumin protein is associated with increased inhibition, whereas loss of the parvalbumin interneurons as a whole is associated with decreased inhibition (Wohr et al., 2015; Filice et al., 2016).

It should be noted that a number of models of hypoxic-ischemic perinatal injury have also shown changes in parvalbumin interneurons and, in some cases, their perineuronal nets. In the recent study by Fowke et al. (2018), a substantial loss of interneurons was observed in the cortex, and there was an associated disruption of the perineuronal nets within 7 days of the injury, particularly in layer 6 of the cortex. Loss of calbindin and parvalbumin immunopositive neurons from the striatum has also been reported after perinatal asphyxia (Van de Berg et al., 2003). However, as mentioned above, due to the much greater severity of this model of brain injury, compared with the inflammation-injury model used here, the mechanism of damage is likely to be quite different. In the context of severe damage, it is unclear how much the interneuron damage alone contributes to the neuropathology and behavioral deficits observed in cases of human hypoxic-ischemic encephalopathy.

Early changes in calretinin-positive interneurons were also seen in this study, both in human and mouse tissue; however, in mouse, they did not persist after the initial injury period. Calretinin-positive neurons are present in the cortex from as early as 12 weeks of gestation in the human brain (Al-Jaberi et al., 2015), possibly explaining their early response to injury. A reduction in calretinin-positive neurons was also observed in the visual cortex (but not other cortical regions) in a preterm baboon exposed to ventilator support (Verney et al., 2010) but not in the caudate following prenatal cerebral ischemia in sheep (McClendon et al., 2014). However, it has recently been shown that there is a significant decrease in size and number of calretinin-positive neurons in the caudate nucleus in the brains of autistic patients (Adorjan et al., 2017). A previous study of adult males with autism also showed a decrease in calretinin-positive interneurons specifically localized in the dentate gyrus of the hippocampus and more widespread changes in parvalbumin-positive interneuron numbers (Lawrence et al., 2010). Of note, Canetta et al. (2016) clearly show no change in calretinin, or somatostatin, interneurons in their model of maternal immune activation-induced brain injury. This suggests that calretinin and parvalbumin injury may occur *via* different mechanisms, or that early, mild changes in calretinin interneurons are normalized as development progresses.

The altered frequency of synaptic boutons on the dendrites of Golgi stained neurons in the cortex was an interesting finding. Favuzzi et al. (2017) have shown that altering the integrity of perineuronal nets by the deletion of one component, in this case brevican, alters the electrophysical properties of interneurons, and subsequently the number of synapses of parvalbumin interneurons onto pyramidal neurons. While in the present study, there was not a significant loss of perineuronal nets at P40, the time when this altered synaptic distribution was observed, there was a small but significant decrease in the number of cells positive for both parvalbumin and perineuronal nets. This may suggest an on-going reduction in inhibitory control in the cortex and a related change in the pyramidal neuron function. Electrophysiological studies will need to be performed to confirm if this is the case. However, it is important to note that long-term behavioral changes have been observed in this mouse model (Favrais et al., 2011) where animals treated with IL-1β from P1 to P5 fail to recognize novel or displaced objects in memory tests. Brevican deletion in the adult also showed impaired working and short-term memory (Favuzzi et al., 2017) supporting the assumption that the reduction in parvalbumin interneurons and perineuronal nets observed in this study may contribute to a disordered brain function.

In conclusion, this study provides clinically important information on the effects of preterm birth to decrease parvalbumin neurons and their perineuronal nets, disrupt the excitatory-inhibitory cell balance, and reduce spine density in the cortex. Altogether, these suggest that a decrease in cortical inhibition may result from preterm birth and its associated exposures to inflammatory injuries, though the potential contribution of other factors in this complex clinical situation cannot be ruled out. The pathology can be consistently recognized from P10 in the mouse, approximately equivalent to term in humans. This suggests that it may be possible for pharmacological tools that modulate cortical excitability to correct the abnormal developmental trajectory of the brain. This would open therapeutic avenues for reducing the long-term burden of neurodevelopmental sequelae occurring in many millions of infants every year due to brain injury associated with preterm birth.

## Ethics Statement

This study was carried out in accordance with the recommendations of National Health Services (NHS) UK guidelines with written informed consent from all subjects. All subjects gave written informed consent in accordance with the Declaration of Helsinki. The protocol was approved by the National Research Ethics Services (West London), UK (ethics number: 07/H0707/139; Post-mortem Magnetic Imaging Study of the Developing Brain). This study was carried out in accordance with the recommendations of UK Home Office according to the regulations in the Animal (Scientific Procedures) Act (2012). The protocol was approved by the King’s College London (KCL) Animal Welfare and Ethical Review Board, PPL 70/8376.

## Author Contributions

HS, BF, YA, and PG contributed to conceptualization. HS, YA, SB, VS, RV, and AY contributed to formal analysis and investigation. CT, MR, AE, and PG contributed to resources. HS, BF, and AB contributed to data curation and visualization. HS contributed to writing – original draft preparation. All authors contributed to writing – review and editing. HS, CT, BF, MR, AE, and PG contributed to supervision. HS and PG contributed to project administration. HS, PG, CT, MR, and AE contributed to funding acquisition.

### Conflict of Interest Statement

The authors declare that the research was conducted in the absence of any commercial or financial relationships that could be construed as a potential conflict of interest.
